# Updated Trends in Valvular Heart Disease-Related Heart Failure in G20-the Group of Twenty Countries: Insights From the Global Burden of Disease Study 2021

**DOI:** 10.31083/RCM38912

**Published:** 2025-08-29

**Authors:** Cheng Liu, Shenghui Zhang

**Affiliations:** ^1^Department of Cardiology, Guangzhou First People’s Hospital, South China University of Technology, 510180 Guangzhou, Guangdong, China; ^2^Department of Cardiology, Guangzhou First People’s Hospital, Guangzhou Medical University, 510180 Guangzhou, Guangdong, China; ^3^Department of Internal Medicine, The Second Affiliated Hospital of Guangzhou Medical University, 510260 Guangzhou, Guangdong, China

**Keywords:** valvular heart disease, heart failure, disease burden, Group of 20

## Abstract

**Background:**

Valvular heart disease (VHD), including both non-rheumatic valvular heart disease (NRVHD) and rheumatic valvular heart disease (RVHD), is a major global health concern. Moreover, the progression of VHD to heart failure (HF) poses substantial clinical and public health challenges. In light of the global population aging, alongside increasing cardiovascular risk factors, and the additional strain imposed by the COVID-19 pandemic, a timely reassessment of the VHD-related HF burden is urgently needed. Using the most recent data from the Global Burden of Disease (GBD) Study 2021, this study aimed to evaluate the distribution of VHD-related HF burden in 2021, examining the long-term trends from 1990 to 2021, and short-term changes between 2019 and 2021, to provide updated insights to inform future prevention and management strategies.

**Methods:**

Using GBD 2021 data, we analyzed the distribution of VHD-related HF burden in age-standardized prevalence rates across the Group of Twenty (G20) countries.

**Results:**

The highest NRVHD-related HF burden in 2021 was observed in the United States (US), Italy, and Russia, while the highest RVHD-related HF burden was noted in India, France, and China. Over the past 30 years (1990–2021), the NRVHD-related HF burden decreased in developed countries (e.g., the US, Canada, Japan) but increased in emerging economies (e.g., India, Brazil, South Africa), with significant increases also observed in Argentina, Mexico, Brazil, among other countries. Notably, nearly all G20 countries exhibited a downward trend in RVHD-related HF burden, with Germany and Australia being the exceptions. During the COVID-19 pandemic (2019–2021), the NRVHD-related HF burden declined in most G20 nations, except for South Africa, India, and a few others, while the RVHD-related HF burden increased slightly in countries such as Mexico, Russia, and Indonesia.

**Conclusions:**

Trends in NRVHD- and RVHD-related HF burden across G20 countries exhibited notable variations, and these became more pronounced under the impact of the COVID-19 pandemic. These findings underscore the importance of developing long-term strategies to enhance the resilience of healthcare systems, improve chronic disease management, and optimize resource allocation to promote cardiovascular health and preparedness for public health challenges.

## 1. Introduction

Valvular heart disease (VHD), encompassing both non-rheumatic valvular heart disease (NRVHD) and rheumatic valvular heart disease (RVHD), remains a significant global health issue. The increasing burden of NRVHD is largely driven by an aging population, improved healthcare, and longer life expectancy, contributing to a rise in related heart failure (HF). Despite advancements in medical care, RVHD persists in low- and middle-income countries and has even resurged in certain high-income nations [1]. According to the Global Burden of Disease Study (GBD) 2019, both NRVHD and RVHD affected millions globally, with RVHD-related mortality being notably higher. As global population aging, increasing cardiovascular risk factors, and the additional strain imposed by the COVID-19 pandemic, the progression of VHD to HF remains a critical clinical challenge, with current treatments proving inadequate once HF develops. With the release of GBD 2021 data, which incorporates additional data and accounts for the impact of the COVID-19 pandemic, reassessment is crucial to inform targeted prevention and management strategies. This study aims to provide updated insights into the burden and trends of VHD-related HF across the Group of Twenty (G20), analyzing long-term trends (1990–2021) and short-term changes (2019–2021) to guide more targeted prevention and management strategies.

## 2. Methods

Based on previously published methods [1], this study utilized data from the GBD 2021 to assess the burden of VHD-related HF across G20 countries. VHD was categorized into RVHD and NRVHD based on the codes from International Statistical Classification of Diseases and Related Health Problems (10th Revision), with RVHD corresponding to I01-I09.9 and NRVHD to I34-I37.9. VHD-related HF was defined as HF directly caused by valvular abnormalities. VHD-related HF refers to HF that arises as a direct consequence of valvular abnormalities, including RVHD or NRVHD. These categories align with clinicaldiagnostic criteria outlined by the American College of Cardiology/American Heart Association Joint Committeeon Clinical Practice Guidelines. Similar to the study by Tang *et al*. [2] on HF impairment in GBD-2019 study, in this study data on HF impairment with RVHD and NRVHD was collected from the GBD-2019 study according to the following process: (i) Find the GBD Results tools (https://vizhub.healthdata.org/gbd-results/); (ii) Select the “impairment” option in the “GBD Estimate” box; (iii) Select the “heart failure” option in the “Impairment” box, including treated HF, mild HF, moderate HF and severe HF; (iv) Select both the “Rheumatic heart disease” and “Non-rheumatic valvular heart disease” option in the “Cause” box. We analyzed age-standardized prevalence rates (ASPRs) of VHD-related HF for the years 1990, 2019, and 2021 across G20 countries, with data extracted from the GBD 2021 database. To account for uncertainty, 95% uncertainty intervals were generated based on 1000 draws. Temporal trends in ASPRs from 1990 to 2021 and short-term changes from 2019 to 2021 were assessed using estimated annual percentage change, calculated via least squares linear regression. Data analysis was performed using R (version 4.2.1, R Foundation for Statistical Computing, Vienna, Austria) and Origin Pro 2024 (OriginLab Corporation, Northampton, MA, USA).

## 3. Results

### 3.1 VHD-Related HF Burden in G20 Countries in 2021

**Fig. 1.**
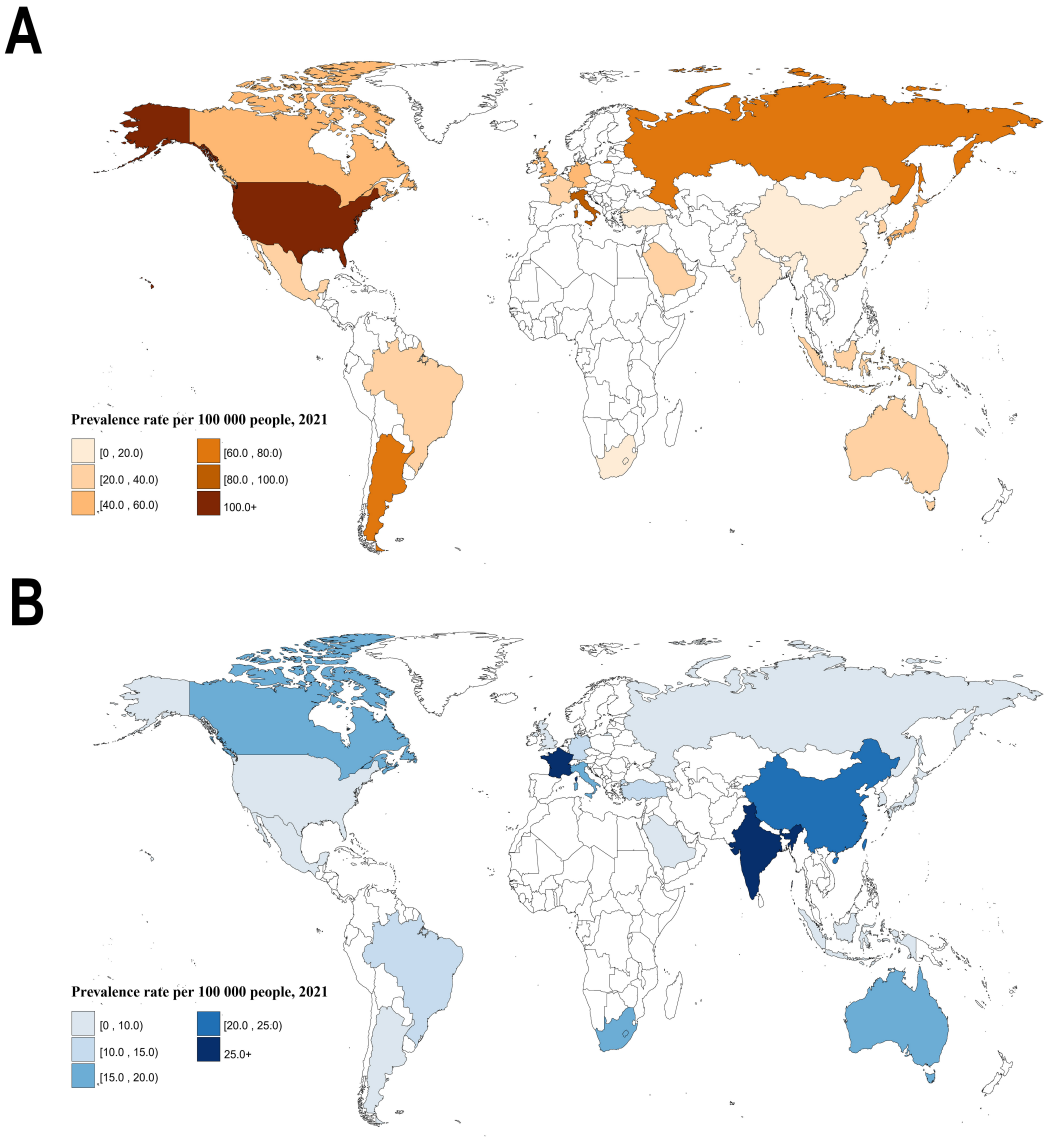
**The ASPRs of NRVHD- and RVHD-related HF burden among G20 countries in 2021**. (A) The ASPRs of NRVHD-related HF burden among G20 countries in 2021. (B) The ASPRs of RVHD-related HF burden among G20 countries in 2021. Note: ASPRs, age-standardized prevalence rates; NRVHD, non-rheumatic valvular heart disease; RVHD, rheumatic valvular heart disease; HF, heart failure; G20, the Group of Twenty.

As shown in Fig. 1A and **Supplementary Table 1**, the seven G20 countries with the highest NRVHD-related HF burden in 2021 were the United States, Italy, Russia, Argentina, the United Kingdom, Germany, and Japan, with ASPRs all greater than 50 per 100,000 person-years. The United States had the highest burden at 101.86 per 100,000 person-years. In contrast, the seven countries with the lowest burden were South Africa, India, Turkey, China, Saudi Arabia, Indonesia, and Brazil, with ASPRs all less than 30 per 100,000 person-years, with South Africa having the lowest at 5.6 per 100,000 person-years. Compared to the GBD-2019 data [1], the changes in 2021 were as follows: (I) The NRVHD-related HF burden increased in most G20 countries, particularly in Germany, France, Argentina, Brazil, and Mexico, with Argentina showing the most significant increase; (II) Italy and China were among the few countries where the burden decreased. Although Italy’s burden declined, it remained relatively high. The United States surpassed Italy to become the country with the highest NRVHD-related HF burden.

Meanwhile, as shown in Fig. 1B and **Supplementary Table 1**, the highest RVHD-related HF burden among the G20 countries in 2021 was observed in India, France, China, South Africa, Italy, Australia, and Canada, all with ASPRs exceeding 15 per 100,000 person-years. India had the highest burden at 64.46 per 100,000 person-years. In contrast, the countries with the lowest burden included the United Kingdom, Russia, Saudi Arabia, Argentina, South Korea, Mexico, and Japan, all with ASPRs below 7 per 100,000 person-years, with the United Kingdom reporting the lowest at 4.52 per 100,000 person-years. Compared to the updated GBD-2019 data [1], emerging economies such as India, Brazil, Turkey, and Indonesia, along with developed nations like France and Germany, experienced an increase in RVHD-related HF burden. Notably, India surpassed China to become the country with the highest burden. Conversely, China, Italy, South Africa, and the United Kingdom saw a decline, with China and Italy exhibiting particularly significant reductions, although their burden remained relatively high. These trends may be attributed to both the persistence of existing RVHD burden and the emergence of new cases. In regions such as India and South Africa, where rheumatic fever and streptococcal infections remain prevalent, the limited availability of preventive measures and early diagnostic resources, particularly in rural and resource-constrained areas, could be contributing factors.

### 3.2 Long-Term Changes (1990–2021) in VHD-Related HF Burden Among G20 Countries

**Fig. 2.**
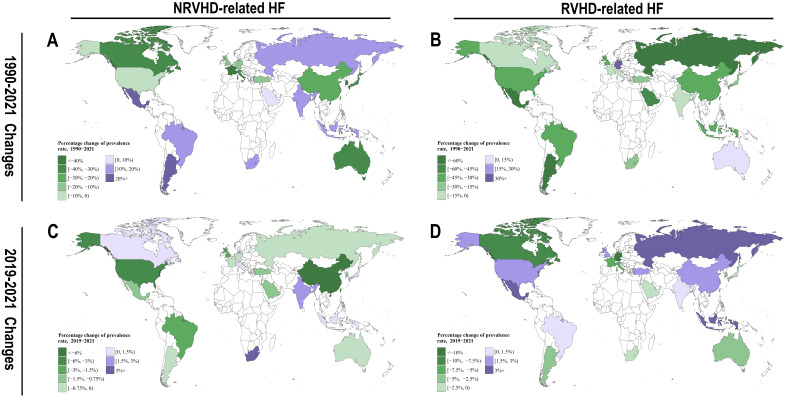
**The long-term (1990–2021) and short-term (2019–2021) Changes on ASPRs of NRVHD- and RVHD-related HF burden among G20 countries**. (A) The long-term (1990–2021) Changes on ASPRs of NRVHD-related HF burden among G20 countries. (B) The long-term (1990–2021) Changes on ASPRs of RVHD-related HF burden among G20 countries. (C) The short-term (2019–2021) Changes on ASPRs of NRVHD-related HF burden among G20 countries. (D) The short-term (2019–2021) Changes on ASPRs of RVHD-related HF burden among G20 countries. Note: ASPRs, age-standardized prevalence rates; NRVHD, non-rheumatic valvular heart disease; RVHD, rheumatic valvular heart disease; HF, heart failure; G20, the Group of Twenty.

As illustrated in Fig. 2A and **Supplementary Table 2**, over the past 30 years, the burden of NRVHD-related HF generally declined in developed countries, including the United States, Canada, the United Kingdom, France, Italy, Germany, Japan, and South Korea. In contrast, emerging economies such as India, Brazil, Mexico, Indonesia, Russia, and South Africa experienced an increasing trend. The most significant rises in burden occurred in Argentina, Mexico, Brazil, Indonesia, South Africa, Russia, and India, with increases exceeding 10%. Conversely, South Korea, France, Canada, Japan, Italy, Australia, and China saw the most substantial reductions, each surpassing 20%.

Similarly, as depicted in Fig. 2B and **Supplementary Table 2**, the burden of RVHD-related HF exhibited a downward trend across nearly all G20 countries. The most notable reductions were recorded in Mexico, Russia, Argentina, Saudi Arabia, the United Kingdom, Brazil, the United States, Indonesia, China, Japan, and South Africa, with declines exceeding 25%. This progress was largely attributed to advancements in prevention and management of rheumatic heart disease, including early antibiotic treatment for streptococcal infections, improved sanitation, and measures to prevent recurrent rheumatic fever. Notably, Germany and Australia were the only two G20 countries where the RVHD-related HF burden increased over this period, with Germany experiencing the most significant rise-approximately six times that of Australia. This trend was likely linked to a rising RVHD burden in specific regions or high-risk populations, such as immigrant communities [3, 4]. A similar pattern was observed in other European countries with relatively lenient immigration policies, including France and Italy [5].

### 3.3 Short-Term Changes (2019–2021) in VHD-Related HF Burden Among G20 Countries

During the COVID-19 pandemic, changes in the VHD-related HF burden across G20 countries varied significantly. The key findings were as follows: (I) As shown in Fig. 2C and **Supplementary Table 2**, the NRVHD-related HF burden declined in most countries, except for South Africa, India, Canada, Italy, Indonesia, and Japan. Notably, China and the United States experienced the most significant reductions, followed by Brazil and the United Kingdom. In contrast, South Africa recorded the largest increase. (II) As shown in Fig. 2D and **Supplementary Table 2**, the RVHD-related HF burden slightly increased in some countries during the pandemic, particularly in Mexico, Russia, Indonesia, Turkey, and China. However, in other countries, this burden declined, with Germany experiencing the most significant reduction, followed by Canada, France, and Italy.

## 4. Discussion

In contrast to the long-term trends, short-term trends revealed a mix of continuations and reversals. The following phenomena were observed:

### 4.1 2019–2021 Changes in Countries With Decreased NRVHD- and RVHD-Related HF Burden Based on GBD-2019 Data 

From 1990 to 2019, countries like the United States, Canada, China, Japan, South Korea, the United Kingdom, France, Italy, and Turkey saw decreases in both NRVHD and RVHD-related HF burden. However, between 2019 and 2021, the trends followed three main patterns: (A) South Korea and France continued to experience reductions in both NRVHD and RVHD-related HF burden. Despite the challenges posed by the COVID-19 pandemic, their cardiovascular disease prevention and treatment systems remained resilient, demonstrating the effectiveness of their heart health strategies. Korea’s success in managing the COVID-19 pandemic can be attributed to its long-term investment in public health infrastructure, its ability to adapt based on past experiences, and its strong financing mechanisms [6, 7]. Unlike South Korea, France implemented the “France Relance Recovery Plan” during the COVID-19 epidemic, aiming to strengthen prevention [8]. Recent study by Minka *et al*. [8] reported that the HF incidence in France did decrease during the pandemic [9]. (B) Japan, Canada, and Italy saw continued declines in RVHD-related HF burden but an increase in NRVHD-related HF burden, reflecting the negative impact of the pandemic on chronic disease management, despite being developed nations. During the pandemic, the implementation of public health measures against COVID-19 inadvertently contributed to a reduced incidence of rheumatic fever, thereby alleviating the burden of RVHD-related HF [10]. On the other hand, although the HF treatment models in Japan, Canada, and Italy differed, all experienced varying degrees of treatment disruption during the pandemic [11, 12], similarly due to public health control measures. These included limited use of life-saving medications such as sacubitril/valsartan [13] and delays in performing valve surgeries [14], which contributed to an increased burden of NRVHD-related HF. (C) The United States, China, the United Kingdom, and Turkey saw reductions in NRVHD-related HF burden, but a slight rise in RVHD-related HF burden. Notably, China and the United States experienced the most significant reductions in NRVHD-related HF burden among G20 countries, highlighting the effectiveness of their public health policies during the pandemic, such as China’s strict anti-contagion policies [15]. As countries with relatively well-developed healthcare systems and abundant medical resources, China and the United States were able to respond promptly to the COVID-19 pandemic. Although routine management of chronic conditions related to NRVHD was delayed during the early stages of the pandemic [16, 17], these services were subsequently restored. Both countries promptly adapted their HF management strategies [18], ensuring continued access to evidence-based medications such as sacubitril/valsartan and sodium-glucose cotransporter 2 inhibitors [19]. Moreover, the presence of well-established telemedicine platforms and community support systems helped NRVHD patients maintain essential care during the pandemic [20]. However, due to the substantial reallocation of medical resources toward combating COVID-19, the management of RVHD was adversely affected. In addition, existing RVHD patients faced an increased risk of HF readmission following COVID-19 infection, further contributing to the burden of RVHD-related HF [1, 21].

### 4.2 2019–2021 Changes in Countries With Decreased NRVHD- and Increased RVHD-Related HF Burden Based on GBD-2019 Data

Between 1990 and 2019, both Germany and Australia experienced a decrease in NRVHD-related HF burden, while RVHD-related HF burden increased. However, from 2019 to 2021, the NRVHD-related HF burden continued to decline (or remained relatively stable), while the RVHD-related HF burden also decreased. These trends indicate that the healthcare systems in both countries demonstrated remarkable adaptability and effective strategies in responding to public health emergencies, successfully reallocating resources to reduce the burden of chronic diseases.

In the case of Germany, several key factors contributed to this positive outcome. First, Germany made significant strides in managing non-rheumatic HF, particularly by addressing high-risk factors such as hypertension and diabetes. These advancements were driven by improvements in healthcare infrastructure, disease prevention programs (including better management of HF risk factors), and more effective treatment outcomes. Over the past 30 years, Germany has seen an increase in aortic and mitral valve surgeries, facilitated by technological advancements, greater accessibility, and improved patient prognosis [22, 23, 24], clearly outperforming France [25]. Even during the pandemic, although valve surgeries were delayed in the initial phase, the delay was less significant during the second wave of COVID-19 [26]. Furthermore, Germany’s reduction in RVHD-related HF burden during the pandemic reflects the country’s strong healthcare capacity to manage rheumatic HF. In the early stages of the pandemic, treatment options for HF-such as pharmacotherapy, cardiac resynchronization therapy, and valve surgeries-remained largely unaffected [27]. While the pandemic temporarily disrupted the prescription and implementation of new HF medications, Germany quickly adopted the combined treatment of sacubitril/valsartan and Sodium-glucose Cotransporter-2 Inhibitors after the European Society of Cardiology released updated guidelines. This rapid adaptation underscores the critical importance of promoting guideline-directed HF management and bolstering healthcare system resilience during public health crises [28]. Germany’s success in managing the VHD-related HF burden offered valuable lessons for other countries.

Additionally, like South Africa, Australia developed its own national guidelines for RVHD control in response to the rising burden of RVHD [29].

### 4.3 2019–2021 Changes in Countries With Increased NRVHD- and Decreased RVHD-Related HF Burden based on GBD-2019 Data

Between 1990 and 2019, countries such as Mexico, Brazil, Argentina, Indonesia, India, the Russian Federation, South Africa, and Saudi Arabia saw an increase in NRVHD-related HF burden and a decrease in RVHD-related HF burden. However, from 2019 to 2021, the burden trends followed three key shifts: (A) Mexico, Brazil, and Russia experienced a reversal, with NRVHD-related HF burden decreasing and RVHD-related HF burden increasing, likely due to pandemic-induced healthcare challenges. It is also worth noting that from 1990 to 2021, Germany saw a significant increase in RVHD-related HF burden, making it the G20 country with the largest rise, while Mexico experienced the most substantial reduction. However, this trend reversed between 2019 and 2021. The increase in RVHD-related HF burden in Germany from 1990 to 2021 can be attributed to the growing burden of RVHD among potentially high-risk populations, such as immigrant groups. A similar trend has also been observed in other European countries, including France and Italy [1]. In contrast, the reduction in RVHD-related heart failure burden in Mexico, Brazil, and across Latin America can be attributed to several key factors: (1) the active implementation of prevention and screening programs [30], which achieved higher coverage rates, thereby contributing to a decline in RVHD incidence [31]; (2) phenotypic studies of acute HF in Latin America, which identified VHD as the second leading cause of acute HF hospitalization, following coronary artery disease. A relatively high proportion of HF patients had received guideline-directed therapy [32]; and (3) RVHD continued to be the leading etiology for valvular surgery in the region [33], with valvular intervention practices becoming more standardized and making notable progress [34]. Similar experiences had been reported in Russia [35, 36]. South American countries, such as Mexico and Brazil, must urgently adopt more effective policies and resource allocation strategies, drawing on the experiences of Germany’s healthcare system, to effectively tackle the dual challenges posed by chronic diseases and public health emergencies [37]. (B) In Argentina and Saudi Arabia, the RVHD-related HF burden continued to decrease, while NRVHD-related HF burden saw a brief decline, indicating a shift toward NRVHD-related HF burden. As the population ages of the two countries, the incidence of NRVHD has been steadily increasing, further exacerbating the burden [38, 39]. (C) South Africa, Indonesia and India witnessed an increased NRVHD-related HF burden alongside a rise or no change in RVHD-related HF burden, reflecting the dual challenge of rising non-communicable disease burden and inadequate control of infectious disease-related HF [40, 41].

## 5. Limitations

One important limitation of this study was that the GBD database did not provide detailed information on specific types or severity of VHD (e.g., calcific aortic stenosis vs. rheumatic mitral stenosis), nor did it allow differentiation between cases where valvular pathology was the primary cause of HF and those where it was a comorbid condition. Moreover, not all individuals with VHD developed HF, and HF itself could result from a wide range of non-valvular causes. Therefore, the findings should be interpreted as reflecting population-level burden and associations, rather than direct causal relationships between VHD and HF. Another limitation was that, due to variability in healthcare infrastructure, diagnostic capacity, and access to medical care across countries, observed differences in valvular heart disease burden may have partially reflected disparities in disease recognition and reporting, rather than true epidemiological differences. As such, interpretations should have been made with caution and were, to some extent, speculative.

## 6. Conclusions

This study provides updated and comprehensive insights into the evolving burden of VHD-related HF across G20 countries, utilizing the latest GBD 2021 data. Our findings revealed the trends in NRVHD- and RVHD-related HF burden across G20 countries exhibited notable variations, and these became more pronounced under the impact of the COVID-19 pandemic. The data underscore the urgent need for country-specific strategies. For countries with persistently high or rising NRVHD-related HF burden, especially in the context of aging populations, proactive strategies focused on early detection, risk factor control, and equitable healthcare access are essential. In contrast, countries where RVHD remains a concern require context-specific approaches. In some countries, the RVHD-related HF burden largely reflected a legacy of previously diagnosed cases, calling for continued long-term management and surveillance to prevent complications. In others, the RVHD burden is driven by ongoing new cases, emphasizing the urgent need to strengthen primary prevention strategies, including rheumatic fever control, early detection, and timely antibiotic prophylaxis in vulnerable populations.

The pandemic further highlighted disparities in healthcare system resilience. Countries that managed to stabilize or reduce the VHD-related HF burden during this period often benefited from robust chronic disease management systems, well-developed healthcare infrastructure, and timely public health interventions. Their successful experiences provided valuable insights and warranted broader dissemination. These findings emphasized the need for sustained investments in strengthening health systems, maintaining continuity of care for chronic conditions, and developing adaptable response mechanisms to better cope with future public health emergencies.

Overall, the data highlight the recent changes in the burden of VHD-related HF across G20 countries and the impact of the COVID-19 pandemic. The findings emphasize the significant role of chronic disease management, healthcare resource allocation, and public health policies in addressing these health challenges. Additionally, they provide important insights for the ongoing management of VHD-related HF burden in the post-pandemic era.

## Availability of Data and Materials

The data for this article comes from the public domain: Institute for Health Metrics and Evaluation (IHME), at https://vizhub.healthdata.org/gbd-results/, accessed on 2 November 2024. All data generated or analyzed during this study are included in this published article.

## Supplementary Material

Supplementary material associated with this article can be found, in the online version, at https://doi.org/10.31083/RCM38912.

## References

[1] Zhang S, Liu C, Wu P, Li H, Zhang Y, Feng K (2024). Burden and Temporal Trends of Valvular Heart Disease-Related Heart Failure From 1990 to 2019 and Projection Up to 2030 in Group of 20 Countries: An Analysis for the Global Burden of Disease Study 2019. *Journal of the American Heart Association*.

[2] Tang H, Zhang N, Deng J, Zhou K (2024). Changing trends in the prevalence of heart failure impairment with Thalassemias over three decades. *European Journal of Clinical Investigation*.

[3] Kötter I, Krusche M (2023). Inflammatory rheumatic diseases in migrants. *Die Innere Medizin*.

[4] Krüger C (2017). Rheumatic Heart Disease Still Relevant. *Deutsches Arzteblatt International*.

[5] Ojha U, Marshall DC, Salciccioli JD, Al-Khayatt BM, Hammond-Haley M, Goodall R (2024). Temporal trend analysis of rheumatic heart disease burden in high-income countries between 1990 and 2019. *European Heart Journal. Quality of Care & Clinical Outcomes*.

[6] Chang RH, Shie WC, Hsu YH, Hur H (2023). Anticipation or resilience in times of emergent crisis? COVID-19 responses in the United States, Taiwan, and South Korea. *Journal of Emergency Management*.

[7] Kim YK, Lee J, Yang Y, Min GY, Shaw R, Pal I (2022). Risk governance, resilience, and response against COVID-19 in the Republic of Korea. *Pandemic Risk, Response, and Resilience*.

[8] Minka SO, Minka FH, Chauvin A, Revue E, Plaisance P, Casalino E (2021). Resilience strategy in emergency medicine during the Covid-19 pandemic in Paris. *Journal Européen des Urgences et de Réanimation*.

[9] Olié V, Isnard R, Pousset F, Grave C, Blacher J, Gabet A (2025). Epidemiology of hospitalized heart failure in France based on national data over 10 years, 2012-2022. *ESC Heart Failure*.

[10] Kaya Akca U, Atalay E, Cuceoglu MK, Balik Z, Sener S, Ozsurekci Y (2022). Impact of the COVID-19 pandemic on the frequency of the pediatric rheumatic diseases. *Rheumatology International*.

[11] Aoki T, Sugiyama Y, Mutai R, Matsushima M (2023). Impact of Primary Care Attributes on Hospitalization During the COVID-19 Pandemic: A Nationwide Prospective Cohort Study in Japan. *Annals of Family Medicine*.

[12] Virani SA, Clarke B, Ducharme A, Ezekowitz JA, Heckman GA, McDonald M (2020). Optimizing Access to Heart Failure Care in Canada During the COVID-19 Pandemic. *The Canadian Journal of Cardiology*.

[13] Rosano GMC, Celant S, Olimpieri PP, Colatrella A, Onder G, Di Lenarda A (2022). Impact of the COVID-19 pandemic on prescription of sacubitril/valsartan in Italy. *European Journal of Heart Failure*.

[14] Prosperi-Porta G, Nguyen V, Eltchaninoff H, Dreyfus J, Burwash IG, Willner N (2024). Impact of the coronavirus disease 2019 pandemic on aortic valve replacement and outcomes in France. *Archives of Cardiovascular Diseases*.

[15] Qi J, Zhang D, Zhang X, Takana T, Pan Y, Yin P (2022). Short- and medium-term impacts of strict anti-contagion policies on non-COVID-19 mortality in China. *Nature Human Behaviour*.

[16] Hirschfeld CB, Shaw LJ, Williams MC, Lahey R, Villines TC, Dorbala S (2021). Impact of COVID-19 on Cardiovascular Testing in the United States Versus the Rest of the World. *JACC. Cardiovascular Imaging*.

[17] Xiang D, Xiang X, Zhang W, Yi S, Zhang J, Gu X (2020). Management and Outcomes of Patients With STEMI During the COVID-19 Pandemic in China. *Journal of the American College of Cardiology*.

[18] Bai F, Pu J, Che W, Chen J, Chen M, Chen W (2023). 2023 Chinese expert consensus on the impact of COVID-19 on the management of cardiovascular diseases. *Cardiology Plus*.

[19] Vaduganathan M, Li D, Van Meijgaard J, Warraich HJ (2021). Prescription Filling Patterns of Evidence-Based Medical Therapies for Heart Failure During the COVID-19 Pandemic in the United States. *Journal of Cardiac Failure*.

[20] Patel SY, Mehrotra A, Huskamp HA, Uscher-Pines L, Ganguli I, Barnett ML (2021). Variation In Telemedicine Use And Outpatient Care During The COVID-19 Pandemic In The United States. *Health Affairs (Project Hope)*.

[21] Tomasoni D, Inciardi RM, Lombardi CM, Tedino C, Agostoni P, Ameri P (2020). Impact of heart failure on the clinical course and outcomes of patients hospitalized for COVID-19. Results of the Cardio-COVID-Italy multicentre study. *European Journal of Heart Failure*.

[22] Frankenstein L, Kaier K, Katus HA, Bode C, Wengenmayer T, von Zur Mühlen C (2021). Impact of the introduction of percutaneous edge-to-edge mitral valve reconstruction on clinical practice in Germany compared to surgical valve repair. *Clinical Research in Cardiology*.

[23] Gaede L, Blumenstein J, Husser O, Liebetrau C, Dörr O, Grothusen C (2021). Aortic valve replacement in Germany in 2019. *Clinical Research in Cardiology*.

[24] Oettinger V, Kaier K, Heidt T, Hortmann M, Wolf D, Zirlik A (2020). Outcomes of transcatheter aortic valve implantations in high-volume or low-volume centres in Germany. *Heart*.

[25] Stachon P, Zehender M, Bode C, von Zur Mühlen C, Kaier K (2018). Development and In-Hospital Mortality of Transcatheter and Surgical Aortic Valve Replacement in 2015 in Germany. *Journal of the American College of Cardiology*.

[26] Heidenreich A, Stachon P, Oettinger V, Hilgendorf I, Heidt T, Rilinger J (2023). Impact of the COVID-19 pandemic on aortic valve replacement procedures in Germany. *BMC Cardiovascular Disorders*.

[27] Bollmann A, Hohenstein S, König S, Meier-Hellmann A, Kuhlen R, Hindricks G (2020). In-hospital mortality in heart failure in Germany during the Covid-19 pandemic. *ESC Heart Failure*.

[28] Kerwagen F, Riemer U, Wachter R, von Haehling S, Abdin A, Böhm M (2023). Impact of the COVID-19 pandemic on implementation of novel guideline-directed medical therapies for heart failure in Germany: a nationwide retrospective analysis. *The Lancet Regional Health*.

[29] Wyber R, Noonan K, Halkon C, Enkel S, Cannon J, Haynes E (2020). Ending rheumatic heart disease in Australia: the evidence for a new approach. *The Medical Journal of Australia*.

[30] Jaimes-Reyes MA, Urina-Jassir M, Urina-Triana M, Urina-Triana M (2022). Current Situation of Acute Rheumatic Fever and Rheumatic Heart Disease in Latin America and the Caribbean: A Systematic Review. *Global Heart*.

[31] Nascimento BR, Sable C, Nunes MCP, Diamantino AC, Oliveira KKB, Oliveira CM (2018). Comparison Between Different Strategies of Rheumatic Heart Disease Echocardiographic Screening in Brazil: Data From the PROVAR (Rheumatic Valve Disease Screening Program) Study. *Journal of the American Heart Association*.

[32] González-Pacheco H, Álvarez-Sangabriel A, Martínez-Sánchez C, Briseño-Cruz JL, Altamirano-Castillo A, Mendoza-García S (2021). Clinical phenotypes, aetiologies, management, and mortality in acute heart failure: a single-institution study in Latin-America. *ESC Heart Failure*.

[33] Zilli AC, Guizilini S, Rocco IS, Santo JADE, Berwanger O, Kalil RAK (2020). Valve Heart Surgery in Brazil - The BYPASS Registry Analysis. *Brazilian Journal of Cardiovascular Surgery*.

[34] Mejia OAV, Borgomoni GB, Palma Dallan LR, Mioto BM, Duenhas Accorsi TA, Lima EG (2022). Quality improvement program in Latin America decreases mortality after cardiac surgery: a before-after intervention study. *International Journal of Surgery*.

[35] Sitnikova MY, Lyasnikova EA, Yurchenko AV, Trukshina MA, Libis RA, Kondratenko VYu (2015). Results of Russian Hospital Chronic Heart Failure Registry in Three Subjects of Russian Federation. *Kardiologiia*.

[36] Yakimenko EA, Zakatova LV, Tbileli VV, Antipova NN, Kolomiets SN, Tikhonchuk NS (2019). Current Trends in the Prevention, Diagnosis and Treatment of Rheumatic Fever and Rheumatic Heart Disease (Review). *Georgian Medical News*.

[37] Armario X, Carron J, Simpkin AJ, Elhadi M, Kennedy C, Abdel-Wahab M (2024). Impact of the COVID-19 Pandemic on Global TAVR Activity: The COVID-TAVI Study. *JACC. Cardiovascular Interventions*.

[38] Ferreiros E, Nacinovich F, Casabé JH, Modenesi JC, Swieszkowski S, Cortes C (2006). Epidemiologic, clinical, and microbiologic profile of infective endocarditis in Argentina: a national survey. The Endocarditis Infecciosa en la República Argentina-2 (EIRA-2) Study. *American Heart Journal*.

[39] Albacker T, Tash A, Alamri H, Alasnag M, Alkashkari W, Almutairi F (2024). Saudi Heart Association/National Heart Center/Saudi Arabian Cardiac Interventional Society/Saudi Society for Cardiac Surgeons/Saudi Cardiac Imaging Group 2023 TAVI Guidelines. *Journal of the Saudi Heart Association*.

[40] Das D, Mohanty S, Guru S, Banerjee A, Kumar A, Deb P (2023). A Retrospective Study on Drug and Dietary Patterns in Patients With Severe Rheumatic Valvular Heart Disease in Eastern India. *Cureus*.

[41] Joseph N, Madi D, Kumar GS, Nelliyanil M, Saralaya V, Rai S (2013). Clinical spectrum of rheumatic Fever and rheumatic heart disease: a 10 year experience in an urban area of South India. *North American Journal of Medical Sciences*.

