# Supplementary material for: Updated Trends in Valvular Heart Disease-Related Heart Failure in G20-the Group of Twenty Countries: Insights From the Global Burden of Disease Study 2021
Source: Rev Cardiovasc Med. 2025 Aug 29;26(8):38912. doi: 10.31083/RCM38912 (PMC12415752; doi:10.31083/RCM38912)
Supplement: Supplementary file 1 [file 2153-8174-26-8-38912-s1.docx]

**Supplementary Table 1.** **The ASPRs of NRVHD- and RVHD- related HF burden among G20 countries in 2021.**

| **Countries** | |  | **Prevalence rate of NRVHD-related HF in 2021** | |  | **Prevalence rate of RVHD-related HF in 2021** | |
| --- | --- | --- | --- | --- | --- | --- | --- |
| **Full name** | **Abbreviated**  **name** |  | **Rank** | **95% UI(per 100,000 person-year)** |  | **Rank** | **95% UI(per 100,000 person-year)** |
| Group of 20 | G20 |  |  | 40.51(25.92 to 62.56) |  |  | 31.24(26.49 to 36.57) |
| European Union | EU |  |  | 56.19(35.80 to 89.35) |  |  | 14.46(12.02 to 17.28) |
| United States of America | USA |  | 1 | 101.86(63.26 to 160.63) |  | 12 | 7.50(5.99 to 9.40) |
| Canada | CAN |  | 8 | 45.10(26.50 to 78.64) |  | 7 | 16.71(13.39 to 20.17) |
| Mexico | MEX |  | 12 | 31.01(19.85 to 48.74) |  | 14 | 6.24(4.79 to 8.12) |
| Brazil | BRA |  | 13 | 29.72(19.32 to 45.81) |  | 9 | 11.31(9.12 to 14.15) |
| Argentina | ARG |  | 4 | 66.72(43.25 to 104.80) |  | 16 | 5.69(4.48 to 7.06) |
| China | CHN |  | 16 | 19.93(12.85 to 30.44) |  | 3 | 23.49(18.18 to 29.93) |
| Japan | JPN |  | 7 | 50.60(30.52 to 82.46) |  | 13 | 6.80(5.45 to 8.32) |
| Republic of Korea | KOR |  | 11 | 33.06(19.78 to 54.18) |  | 15 | 5.90(4.96 to 6.94) |
| Indonesia | IDN |  | 14 | 20.28(13.28 to 30.72) |  | 11 | 9.88(8.41 to 11.71) |
| India | IND |  | 18 | 10.86(7.15 to 16.63) |  | 1 | 64.46(54.92 to 76.12) |
| Russian Federation | RUS |  | 3 | 69.62(44.98 to 107.09) |  | 18 | 4.96(3.60 to 6.83) |
| United Kingdom | GBR |  | 5 | 55.30(33.82 to 88.47) |  | 19 | 4.52(3.50 to 5.67) |
| France | FRA |  | 9 | 36.12(21.73 to 59.41) |  | 2 | 25.39(20.84 to 30.44) |
| Germany | DEU |  | 6 | 53.56(32.84 to 86.21) |  | 8 | 14.29(11.18 to 18.07) |
| Italy | ITA |  | 2 | 86.27(50.15 to 141.62) |  | 5 | 17.33(13.53 to 21.88) |
| Turkey | TUR |  | 17 | 19.64(12.91 to 31.10) |  | 10 | 11.23(9.47 to 13.18) |
| Australia | AUS |  | 10 | 34.41(20.07 to 59.16) |  | 6 | 16.90(14.19 to 19.61) |
| Saudi Arabia | SAU |  | 15 | 20.18(13.09 to 31.86) |  | 17 | 5.49(4.56 to 6.58) |
| South Africa | ZAF |  | 19 | 5.60(3.60 to 9.04) |  | 4 | 19.96(15.06 to 26.38) |

NOTE: ASPRs= age-standardized prevalence rates, HF=heart failure, NRVHD= non-rheumatic valvular heart disease, RVHD= rheumatic valvular heart disease, 95% UI= 95% uncertainty interval.

**Supplementary Table 2. The long-term (1990-2021) and short-term (2019-2021) Changes on ASPRs of NRVHD- and RVHD-related HF burden among G20 countries.**

| **Countries** | |  | **Prevalence rate of NRVHD-related HF burden** | | |  | **Prevalence rate of RVHD-related HF burden** | | |
| --- | --- | --- | --- | --- | --- | --- | --- | --- | --- |
| **Full name** | **Abbreviated name** |  | **Long-term Changes (95% UI)/ (%)^a^** |  | **Short-term Changes  (95% UI)/ (%)^b^** |  | **Long-term Changes (95% UI)/ (%)^a^** |  | **Short-term Changes  (95% UI)/ (%)^b^** |
| Group of 20 | G20 |  | -27.64(-35.76 to -20.27) |  | -3.49(-4.70 to -2.45) |  | -8.05(-12.17 to -3.62) |  | 1.07(-2.62 to 4.72) |
| European Union | EU |  | -21.51(-35.01 to -8.73) |  | -0.72(-3.90 to 1.75) |  | -13.02(-19.91 to -6.07) |  | -7.21(-14.51 to -0.69) |
| United States of America | USA |  | -8.67(-17.76 to -0.11) |  | -4.35(-6.93 to -2.03) |  | -37.78(-45.49 to -28.49) |  | 1.89(-5.22 to 10.79) |
| Canada | CAN |  | -36.71(-47.79 to -23.71) |  | 1.34(-7.93 to 12.29) |  | -7.79(-20.12 to 8.32) |  | -7.79(-16.8 to 1) |
| Mexico | MEX |  | 23.04(16.48 to 29.76) |  | -1.41(-3.01 to 0.09) |  | -74.53(-77.72 to -70.56) |  | 4.31(-3.55 to 13.15) |
| Brazil | BRA |  | 18.37(11.3 to 26.13) |  | -2.76(-4.57 to -0.51) |  | -43.06(-48.54 to -36.42) |  | 1.18(-4.37 to 7.54) |
| Argentina | ARG |  | 23.63(10.43 to 41.05) |  | -0.12(-9.52 to 10.52) |  | -63.21(-67.78 to -58.1) |  | -2.74(-11.72 to 7.16) |
| China | CHN |  | -22.51(-33.25 to -11.63) |  | -7.49(-8.74 to -6.42) |  | -31.83(-39.52 to -23.3) |  | 2.8(-4.17 to 9.82) |
| Japan | JPN |  | -34.16(-44.94 to -24.87) |  | 0.11(-0.89 to 1.21) |  | -28.09(-37.15 to -17.28) |  | -0.43(-6.42 to 5.85) |
| Republic of Korea | KOR |  | -52.99(-66.13 to -38.36) |  | -0.82(-10.89 to 9.55) |  | -7.96(-17.52 to 3.55) |  | -2.87(-10.8 to 5.76) |
| Indonesia | IDN |  | 16.88(13.31 to 20.43) |  | 0.83(-0.64 to 2.31) |  | -36.07(-40.98 to -30.88) |  | 3.06(-1.26 to 7.51) |
| India | IND |  | 11.48(7.99 to 15.72) |  | 1.90(0.95 to 2.91) |  | -12.07(-16.12 to -7.93) |  | 0.76(-2.82 to 4.23) |
| Russian Federation | RUS |  | 12.44(0.96 to 23.06) |  | -0.06(-0.95 to 0.91) |  | -66.77(-72.74 to -59.41) |  | 3.16(-5.73 to 11.74) |
| United Kingdom | GBR |  | -10.35(-23.16 to 1.61) |  | -2.61(-4.21 to -1.32) |  | -43.44(-49.37 to -37.05) |  | 1.94(-4.57 to 9.88) |
| France | FRA |  | -41.73(-54.59 to -27.11) |  | -0.20(-7.56 to 7.51) |  | -3.77(-17.87 to 11.2) |  | -5.27(-18.9 to 6.89) |
| Germany | DEU |  | -16.64(-35.83 to 1.26) |  | -0.10(-11.23 to 11.77) |  | 37.01(20.76 to 55.52) |  | -20.21(-33.04 to -6.47) |
| Italy | ITA |  | -31.98(-45.23 to -19.93) |  | 1.09(-0.15 to 2.24) |  | -3.37(-16.3 to 10.27) |  | -5.2(-12.9 to 3.64) |
| Turkey | TUR |  | -13.4(-25.41 to 1.03) |  | -1.17(-9.67 to 8.5) |  | -17.29(-23.47 to -10.22) |  | 2.9(-4.45 to 12.18) |
| Australia | AUS |  | -30.76(-44.69 to -16.12) |  | -0.28(-10.29 to 9.89) |  | 6.22(-5.13 to 18.68) |  | -4.84(-13.34 to 3.08) |
| Saudi Arabia | SAU |  | 5.03(-7.07 to 20.62) |  | -1.09(-9.14 to 7.55) |  | -51.89(-57.89 to -45.98) |  | -1.76(-8.24 to 5.02) |
| South Africa | ZAF |  | 12.58(6.92 to 18.95) |  | 5.71(3.53 to 8.15) |  | -26.28(-30.15 to -22.12) |  | -0.09(-3.23 to 2.97) |

NOTE: ASPRs= age-standardized prevalence rates, HF=heart failure, NRVHD= non-rheumatic valvular heart disease, RVHD= rheumatic valvular heart disease,95% UI= 95% uncertainty interval.
^a^ The 1990-2021 was defined as the percentage change in the age-standardized rate of prevalence for NRVHD- and RVHD-related HF burden during the years 1990-2021. The formula was as follows: [(the mean value in 2021 / the mean value in 1990) - 1] * 100. A negative value of Delta represented a decreasing trend of burden, while positive ones indicated an increasing trend.
^b^ The 2019-2021 was defined as the percentage change in the age-standardized rate of prevalence for NRVHD- and RVHD-related HF burden during the years 2019-2021. The formula was as follows: [(the mean value in 2021 / the mean value in 2019) - 1] * 100. A negative value of Delta represented a decreasing trend of burden, while positive ones indicated an increasing trend.
